# Modulation Effect of Peroxisome Proliferator-Activated Receptor Agonists on Lipid Droplet Proteins in Liver

**DOI:** 10.1155/2016/8315454

**Published:** 2015-12-07

**Authors:** Yun-Xia Zhu, Ming-Liang Zhang, Yuan Zhong, Chen Wang, Wei-Ping Jia

**Affiliations:** ^1^Department of Geriatrics, Shanghai Jiao Tong University Affiliated Sixth People's Hospital, 600 Yishan Road, Shanghai 200233, China; ^2^Department of Endocrinology and Metabolism, Shanghai Jiao Tong University Affiliated Sixth People's Hospital, 600 Yishan Road, Shanghai 200233, China; ^3^Shanghai Key Laboratory of Diabetes Mellitus, 600 Yishan Road, Shanghai 200233, China; ^4^Shanghai Diabetes Institute, Shanghai Jiao Tong University Affiliated Sixth People's Hospital, 600 Yishan Road, Shanghai 200233, China

## Abstract

Peroxisome proliferator-activated receptor (PPAR) agonists are used for treating hyperglycemia and type 2 diabetes. However, the mechanism of action of these agonists is still under investigation. The lipid droplet-associated proteins FSP27/CIDEC and LSDP5, regulated directly by PPAR*γ* and PPAR*α*, are associated with hepatic steatosis and insulin sensitivity. Here, we evaluated the expression levels of FSP27/CIDEC and LSDP5 and the regulation of these proteins by consumption of a high-fat diet (HFD) or administration of PPAR agonists. Mice with diet-induced obesity were treated with the PPAR*γ* or PPAR*α* agonist, pioglitazone or fenofibrate, respectively. Liver tissues from *db/db* diabetic mice and human were also collected. Interestingly, FSP27/CIEDC was expressed in mouse and human livers and was upregulated in obese C57BL/6J mice. Fenofibrate treatment decreased hepatic triglyceride (TG) content and FSP27/CIDEC protein expression in mice fed an HFD diet. In mice, LSDP5 was not detected, even in the context of insulin resistance or treatment with PPAR agonists. However, LSDP5 was highly expressed in humans, with elevated expression observed in the fatty liver. We concluded that fenofibrate greatly decreased hepatic TG content and FSP27/CIDEC protein expression in mice fed an HFD, suggesting a potential regulatory role for fenofibrate in the amelioration of hepatic steatosis.

## 1. Introduction

Nonalcoholic fatty liver disease (NAFLD), one of the most common liver diseases worldwide, encompasses a spectrum of liver conditions, ranging from simple steatosis, also called simple fatty liver (SFL), to nonalcoholic steatohepatitis (NASH), advanced fibrosis, and cirrhosis [1]. NAFLD has become a major health concern, with as many as 20%–40% of the general population in western countries and 5%–40% of the general population in countries in the Asia-Pacific region affected by NAFLD [2, 3]. Patients with NAFLD are at significantly higher risk for the development of type 2 diabetes (T2D) and cardiovascular disease [4]. Thus, unraveling the pathogenesis of NAFLD and investigating effective treatment options are essential.

As the most benign form of NAFLD, SFL is characterized by excessive lipid accumulation, mainly in the form of lipid droplets (LDs) in hepatocytes. Structurally, LDs consist of a neutral lipid core surrounded by a phospholipid monolayer and proteins embedded in or bound to the phospholipid layer, namely, LD-associated proteins (LDAPs). Importantly, LDs are now recognized not merely as a static neutral lipid storage site but instead as multifunctional organelles involved in lipid metabolism and transport, intracellular trafficking, signaling, and cytoskeletal organization [5]. LDAPs are crucial for LD formation, growth, transport, and hydrolysis and play key roles in various functions of LDs [6]. Most importantly, increasing evidence has shown that there is a relationship between LDAPs and lipid metabolism in hepatocytes of rodents and humans [7]. Fat-specific protein 27 (FSP27)/cell death-inducing DFF45-like effector-C (CIDEC) and lipid storage droplet protein 5 (LSDP5), two members of the LDAP family of proteins, have been shown to facilitate liver steatosis and regulate insulin sensitivity [8, 9]. Overexpression of FSP27/CIDEC in hepatocytes leads to increased hepatic triglyceride (TG) levels [8], whereas knockout of FSP27/CIDEC in mice induces lean phenotypes [10]. Moreover, FSP27/CIDEC-null mice are resistant to diet-induced obesity and insulin resistance [10], and exposure of primary rat hepatocytes to free fatty acids (FFAs) increases LSDP5 expression and lipid accumulation [9]. Both FSP27/CIDEC and LSDP5 are positively regulated by peroxisome proliferator-activated receptor (PPAR); specifically, LSDP5 is regulated by PPAR*α*, and FSP27/CIDEC is regulated by PPAR*γ* [8, 11, 12]. Thus, activation of either PPAR*γ* or PPAR*α* may lead to upregulation of FSP27/CIDEC or LSDP5, thereby increasing lipid accumulation. Fenofibrate, a PPAR*α* agonist, and pioglitazone, a PPAR*γ* agonist, are widely used in the clinical setting for the management of dyslipidemia and insulin resistance. It is not known whether PPAR activation (fenofibrate and pioglitazone treatment) will increase hepatic lipid content by inducing LADPs expression. If it is true, it would ultimately impair the role of PPAR activators on insulin-sensitizing effects.

In the present study, we used a mouse model of SFL induced by high-fat diet (HFD) to measure the expression of FSP27/CIDEC and LSDP5 in the liver. The expression of FSP27/CIDEC and LSDP5 in liver tissue sample from human with fatty liver was also studied. Moreover, we investigated the effects of PPAR activation on the regulation of these LADPs in HFD-induced obese mice treated with fenofibrate or pioglitazone for 20 weeks. Lastly, the effects of PPAR activators on glucose/lipid metabolism and insulin resistance in HFD mice were also determined.

## 2. Materials and Methods

### 2.1. Animal Study

Male C57BL/6J mice (4 weeks old) were purchased from Shanghai SLAC Laboratory Animal Co. Ltd., China (certificate number: SCXK [Shanghai] 2003-0003), and were housed in rooms with a 12-hour light/dark cycle (lights on 07:00 h). Prior to the dietary and drug manipulation, all mice were provided with standard chow (Shanghai Laboratory Animal Center (SLAC): 55% of energy as carbohydrates, 21% as protein, and 14% as fat) and water* ad libitum*. After 1 week of acclimation, the animals were randomly assigned to receive one of the following treatments for 20 weeks: chow diet, HFD (20% of energy as carbohydrates, 20% as protein, and 60% as fat, as a percentage of total kcal, manufactured by SLAC), HFD + fenofibrate (30 mg/kg body weight, Sigma-Aldrich, St. Louis, MO, USA), and HFD + pioglitazone (10 mg/kg body weight, Sigma-Aldrich). Drugs were administered through a feeding needle once per day; the doses were chosen according to previous studies in which similar doses were used for metabolic studies in mice [13, 14]. Male diabetic C57BL/KsJ* db/db* mice (6 weeks old) were fed a standard chow diet for 12 weeks until they developed spontaneous diabetes. Body weights were measured daily for all mice, and fasting blood samples were collected from the tail vein every 4 weeks. At the end of the study, all animals were fasted for 2 h prior to euthanasia inhalation of isoflurane (Abbott Laboratories, Abbott Park, IL, USA), and livers were surgically collected, frozen in liquid nitrogen, and stored at −80°C for further studies. Adipose tissues were also collected and weighed.

### 2.2. Intraperitoneal Glucose Tolerance Test and Insulin Tolerance Test

After a 1-week acclimation, male C57BL/6J mice were fasted for 10 h before the intraperitoneal glucose tolerance test (GTT) at week 19. After a sample of fasted blood was collected from tail bleeding, animals were given glucose (1 g/kg body weight) by intraperitoneal injection. Blood glucose readings were taken using a glucometer (Freestyle Freedom, Abbott Laboratories) at 0, 30, 60, and 120 min after injection. Insulin tolerance tests (ITT) were carried out 1 week after GTTs. Neutral insulin (1 U/kg body weight, Novo Nordisk, Denmark) was injected intraperitoneally after a 6-hour fast. Blood glucose levels were measured at 0, 15, 30, 60, 90, and 120 min after injection. The areas under the curves for blood glucose in GTT (AUC_g_) and ITT (AUC_itt_) were calculated.

### 2.3. Blood Biochemistry

Serum biochemistry parameters, including triglycerides (TGs), total cholesterol (TC), high-density lipoprotein- (HDL-) cholesterol (HDL-C), and low-density lipoprotein- (LDL-) cholesterol (LDL-C), were measured after overnight fasting in mice. The measurements were performed with a parallel, multichannel analyzer (Glamour 2000, MD Inc., Silicon Valley, California, USA). Serum insulin was determined manually using an enzyme-linked immunosorbent assay (ELISA) kit (Mercodia AB, Sweden).

### 2.4. Human Liver Tissues Collection

The liver tissue samples were collected at the Department of General Surgery (Shanghai Jiao Tong University Affiliated Sixth People's Hospital, Shanghai, China) from man subjects undergoing resection of benign focal hepatic lesions. Samples with hepatitis, cirrhosis, or chronic alcohol use were excluded. The samples were immediately shock-frozen and stored at −80°C. All tissues had been examined by a pathologist who was blinded to the study design. Liver tissues with less than 5% hepatic steatosis were classified into the nonfatty liver (Non-FL) group, while those with more than 20% hepatic steatosis were classified into the fatty liver (FL) group. The clinical background data of both groups were presented in Supplementary Table S1 (see Supplementary Material available online at http://dx.doi.org/10.1155/2016/8315454). The study was approved by the Ethics Committee of Shanghai Jiao Tong University Affiliated Sixth People's Hospital, following the principles of the Declaration of Helsinki. Written informed consent was obtained from each patient.

### 2.5. Analysis of TG Levels in the Liver

For TG measurements, 50 mg liver tissue was homogenized in standard phosphate-buffered saline. Lipids were extracted using a heptane-isopropanol-Tween solution [15]. TG concentrations were measured manually by the enzymatic GPO-PAP method using commercial kits (KHB Instruments, China) and were normalized to sample weight for accurate quantification.

### 2.6. Western Blotting

Liver tissues (50 mg) were ground into a powder under liquid nitrogen, and hepatic protein was extracted by incubation at 4°C in lysis buffer containing protease inhibitors, followed by sonication. The lysates were centrifuged at 12,000 rpm for 20 min, and the supernatants were collected. Protein concentrations were determined by the bicinchoninic acid method. After heating to 95°C for 5 min, the proteins were size-fractionated by sodium dodecyl sulfate polyacrylamide gel electrophoresis (SDS-PAGE) on 10% gels and then transferred to polyvinylidene difluoride membranes (Millipore, MA, USA) at 100 V for 70 min. After washing three times with Tris-buffered saline (TBS), the membranes were blocked with 5% dried nonfat milk (Nestle, China) in TBS-Tween 20 (TBS-T, pH 7.4) and then incubated with appropriate primary antibodies targeting FSP27/CIDEC (PAI-4316, diluted 1 : 1,000, Thermo Fisher Scientific, Loughborough, UK) or LSDP5 (ab63970, diluted 1 : 1,000, Abcam, MA, USA) overnight at 4°C. The membranes were then washed three times with TBS-T for 10 min each and incubated with horseradish peroxidase-labeled donkey anti-rabbit IgG (sc-2313, diluted 1 : 5,000, Santa Cruz Biotechnology, Santa Cruz, CA, USA) in TBS-T for 1 h at room temperature. Membranes were then washed three times for 10 min each in TBS-T and visualized using the enhanced chemiluminescence method (Thermo Scientific Pierce, Rockford, IL, USA). Protein expression values were standardized against *β*-actin protein expression (4697s, Cell Signaling Technology, Danvers, MA, USA). The average intensity for each band was quantified with Image J (NIH, Bethesda, MD, USA).

### 2.7. Statistical Analysis

All data are represented as means ± SEM. For statistical analysis, the differences between groups were examined with one-way analysis of variance followed by Bonferroni's test using SPSS 11.0, and differences with two-tailed *P* values less than 0.05 were considered statistically significant.

## 3. Results

### 3.1. Metabolic Changes Associated with HFD-Induced Obesity in Mice

**Figure 1:**
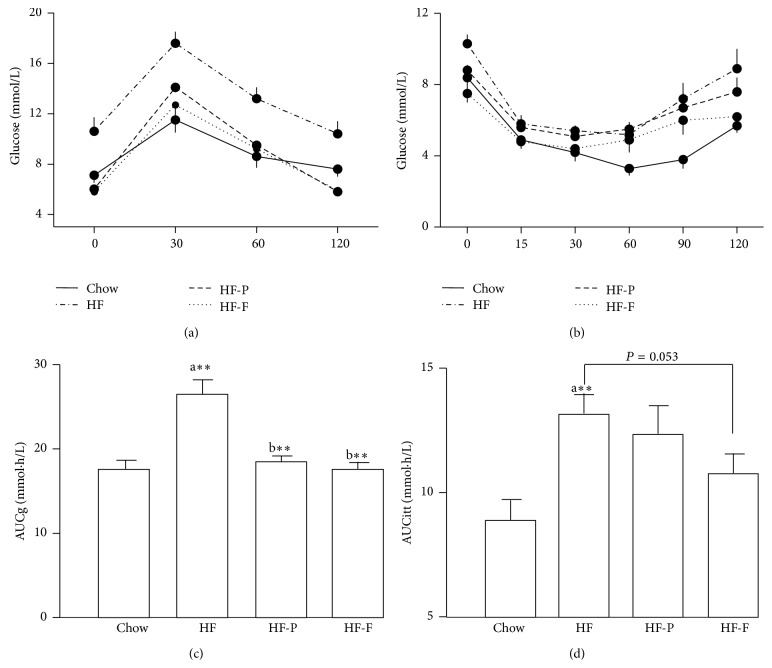
PPAR agonists' treatment improved glucose tolerance and insulin sensitivity in high-fat diet-induced obese mice. Mice were fed with either a normal chow diet (Chow) or a high-fat diet (HF) for 20 weeks and treated with either pioglitazone or fenofibrate. (a) Intraperitoneal glucose tolerance test (GTT), (b) insulin tolerance test (ITT), (c) AUC of GTT, and (d) AUC of ITT. HF-P: mice fed a high-fat diet and treated with pioglitazone; HF-F: mice fed a high-fat diet and treated with fenofibrate. a: versus Chow and b: versus HF; ^*∗∗*^ *P* < 0.01; *n* = 7.

After 20 weeks of consuming an HFD, mice became obese, exhibiting marked increases in body weight, fat mass, glucose level, and TG content in the liver although they did not develop overt diabetes as db/db mice in week 12 (Table 1). In addition, significant changes in serum lipids were observed, as shown by increases in total cholesterol levels (0.77 ± 0.08 versus 1.48 ± 0.14 mM, *P* < 0.01). Mice became profoundly hyperinsulinemic and insulin intolerant, suggesting the acquisition of insulin resistance (Table 1 and Figures 1(b) and 1(d)), and were clearly glucose intolerant (Figures 1(a) and 1(c)).

### 3.2. Effects of PPAR Agonists on HFD-Induced Obesity in Mice

In order to determine the effects of an HFD and treatment with PPAR agonists on metabolism, C57BL/6J mice were treated as described in Section 2, and metabolic parameters were measured. Fenofibrate treatment greatly inhibited the increase in body weight and fat mass (epididymal fat and subcutaneous fat) induced by consumption of an HFD. Fasting glucose levels were reduced early in the experimental period in mice treated with either fenofibrate or pioglitazone. Dyslipidemia was also improved significantly by both PPAR agonists (Table 1). Furthermore, both fenofibrate and pioglitazone corrected glucose tolerance and insulin sensitivity (Figures 1(a)–1(d)). Most importantly, TG levels were reduced in the livers of fenofibrate-treated mice fed an HFD compared with untreated mice fed an HFD (7.5 ± 0.8 versus 12.3 ± 0.7 *μ*mol/g, *P* < 0.01, Table 1).

### 3.3. FSP27/CIDEC and LSDP5 Protein Expression in Liver from HFD-Induced Obese Mice Treated with PPAR Agonists

**Figure 2:**
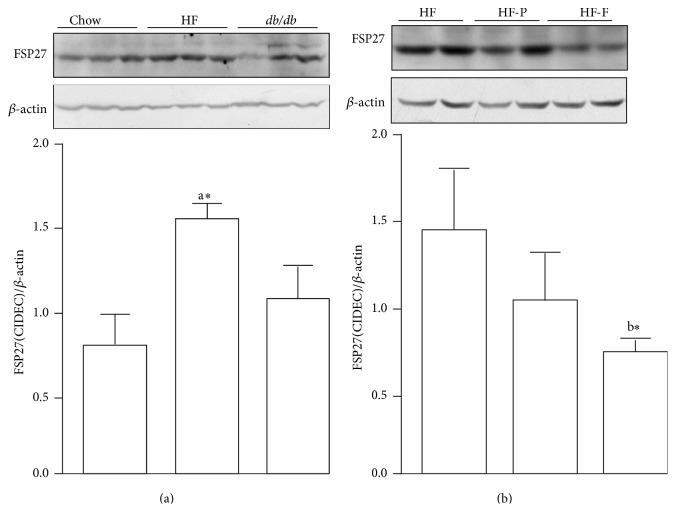
FSP27/CIDEC protein is highly expressed in mouse fatty liver. Immunoblot analyses of FSP27/CIDEC protein abundance in liver lysates (upper panel). Quantification of the protein levels was normalized to *β*-actin (lower panel). (a) FSP27/CIDEC protein level in liver from C57BL/6 mice fed with a normal chow diet (Chow) or high-fat diet (HF) for 20 weeks or from db/db mice. (b) FSP27/CIDEC protein level in liver from mice fed with HF diet and treated with pioglitazone or fenofibrate. Data are the mean ± SEM (*n* = 4–7). a: versus Chow and b: versus HF; ^*∗*^ *P* < 0.05. HF-P: mice fed an HF diet and treated with pioglitazone; HF-F: mice fed an HF diet and treated with fenofibrate.

We further investigated the effects of PPAR activators on the expression of LDAPs. Our results suggested that hepatic FSP27/CIDEC protein expression was significantly enhanced by consumption of an HFD in mice. Compared with mice consuming the standard chow diet, mice fed an HFD exhibited an average 97% increase in FSP27/CIDEC expression (*P* < 0.05, Figure 2(a)). The expected increase in FSP27/CIDEC expression was also observed in the livers of* db/db* diabetic mice, although this difference was not significant (Figure 2(a)). Moreover, after treatment with fenofibrate or pioglitazone daily for 20 weeks, FSP27/CIEDC expression was reduced by 51% and 29%, respectively; this difference was statistically significant for fenofibrate only (*P* < 0.05, Figure 2(b)). Interestingly, LSDP5 expression was nearly undetectable in the livers of C57BL/6J and* db/db* diabetic mice (Supplementary Figure S1).

### 3.4. Expression of FSP27/CIDEC and LSDP5 in Liver Sample from Subjects with Fatty Liver

**Figure 3:**
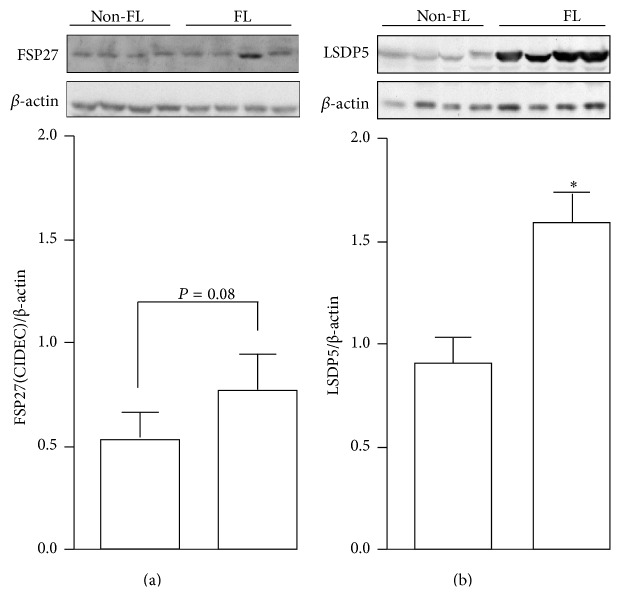
FSP27/CIDEC and LSDP5 protein expression increased in human fatty liver. Immunoblot analyses of FSP27/CIDEC (a) and LSDP5 (b) protein expression (upper panel) from human liver tissue samples with or without fatty liver. Quantification of the protein levels was normalized to *β*-actin (lower panel). Data are the mean ± SEM (*n* = 4). ^*∗*^ *P* < 0.05. Non-FL: subjects without fatty liver and FL: subjects with fatty liver.

In order to validate the association of LDAP expression and hepatic steatosis observed in animal models, we further measured the protein expression of FSP27/CIDEC and LSDP5 in human livers. FSP27/CIDEC expression tended to increase in patients in the FL group compared with patients in the Non-FL group (Figure 3(a)) (*P* = 0.08). Surprisingly, the expression of LSDP5 was significantly higher in patients in the FL group compared with patients in the Non-FL group (1.59 ± 0.15 versus 0.90 ± 0.12, resp., *P* < 0.05, Figure 3(b)).

## 4. Discussion

In the present study, we provided evidence supporting previous findings that both fenofibrate (a PPAR*α* agonist) and pioglitazone (a PPAR*γ* agonist) significantly improve insulin sensitivity and glucose tolerance, with fenofibrate exhibiting superior effects in mice fed an HFD. More importantly, we found for the first time that fenofibrate treatment in mice fed an HFD resulted in a significant decrease in hepatic lipid content, accompanied by reduced FSP27/CIDEC protein expression in the liver. Furthermore, LSDP5 was markedly elevated in liver tissues from patients with FL. These findings indicated that fenofibrate or pioglitazone had no side effects on LSDP5 and FSP27/CIDEC protein expression. In contrast, FSP27/CIDEC may be associated with amelioration of steatohepatitis in the context of long-term fenofibrate treatment. In humans, LSDP5 was positively correlated with hepatic lipid content, consistent with the observations in rodent models.

FSP27/CIDEC belongs to the CIDE family, which includes CIDEA, CIDEB, and CIDEC. All of these proteins contain a conserved CIDE-N domain [15]. CIDEC is the human homolog of mouse FSP27 [16]. CIDE proteins are important regulators of energy homeostasis and are closely linked to the development of metabolic disorders [15]. As a member of the CIDE family, FSP27/CIDEC plays important roles in hepatic steatosis [8, 11, 17, 18]. FSP27/CIDEC is dramatically upregulated in the livers of* ob/ob* and mice with HFD-induced obesity [8, 19]. In vitro and in vivo studies have shown that forced expression of FSP27/CIDEC in hepatocytes leads to an increase in the content of hepatic TGs [8]. Targeted knockdown of FSP27/CIDEC expression in the livers of* ob/ob* mice partially ameliorates FL pathology [8]. Moreover, liver sections from mice injected with adenovirus expressing FSP27 shRNA exhibit smaller and less numerous LDs as compared to mice injected with adenovirus expressing scramble shRNA [8]. Our finding that FSP27/CIDEC was upregulated in FLs from mice fed an HFD was in agreement with the results of a previous report [19]. Moreover, we observed similar results in humans, consistent with a study in patients who underwent gastric bypass surgery, in which hepatic* CIDEC *was significantly downregulated and symptoms of steatosis were ameliorated due to weight loss at 1 year after surgery [17].

FSP27/CIDEC is induced by PPAR*γ* activators and functions as a direct downstream target of hepatic PPAR*γ* [8]. Using a subtractive cloning strategy, researchers have shown that* FSP27* cDNA is specifically expressed in FLs of rosiglitazone-treated liver-specific PPAR*γ*-null (*ob/ob*-PPAR*γ*/C^+^) mice [8]. Additionally, PPAR*γ* is elevated in FLs from murine model of diabetes and obesity [20, 21] and is critical for the development of hepatic steatosis [22, 23]. PPAR*γ* deficiency in the livers of* ob/ob* mice and mice with HFD-induced obesity dramatically improves hepatic steatosis. Indeed, some studies have reported that prolonged treatment of obese and diabetic mice with thiazolidinediones (TZDs, selective PPAR*γ* ligands and activators), including troglitazone, rosiglitazone, and pioglitazone, results in the development of severe hepatic steatosis [24]. Since FSP27/CIDEC is involved in PPAR*γ*-dependent hepatic steatosis [8], it is therefore necessary to determine whether the effects of PPAR*γ* agonists on the formation of FL were mediated by activation of FSP27/CIDEC expression. PPAR*γ* agonists, including troglitazone, rosiglitazone, and pioglitazone, are widely used as insulin sensitizers in the clinical setting. However, troglitazone and rosiglitazone have been withdrawn from the market because of their significant side effects; therefore, only pioglitazone is currently available for clinical use in humans. To our surprise, in the present study, treatment with pioglitazone for 20 weeks neither significantly induced the expression of FSP27/CIDEC nor increased hepatic lipid content in mice with HFD-induced obesity, which was in agreement with a study of alcohol-induced FL [19]. However, Satoh et al. reported that treatment with pioglitazone (9 mg/kg) for 6 weeks exacerbated hepatic steatosis and markedly elevated FSP27 expression in ddY-H mice (a model of spontaneous insulin resistance) fed standard chow [25]. This discrepancy may be explained as follows. First, pioglitazone treatment and consumption of an HFD both activate PPAR*γ* in the liver [26, 27]. Therefore, the induction of FSP27/CIDEC expression by PPAR*γ* may not differ substantially following pioglitazone treatment in mice fed an HFD. Second, given the positive correlation between hepatic TG content and FSP27/CIDEC expression in mice with HFD-induced obesity [19], hepatic FSP27/CIDEC levels may be similar because of the similar hepatic TG contents in mice treated with or without pioglitazone. Third, these studies used different mouse models, which could lead to differences in response to treatment. In the present study, pioglitazone treatment for 20 weeks did not significantly affect hepatic lipid content in mice fed an HFD. However, because these results differ from previous works [28–30], the effects of long-term pioglitazone treatment on hepatic TG content are still unclear. Thus, while it is evident that different models of obesity and diabetes may yield different results, our data from mice fed an HFD supported that long-term pioglitazone treatment may not increase hepatic lipid content in this animal model.

Factors other than PPAR*γ* may also play a role in the regulation of FSP27/CIDEC. Data from our animal research suggested that PPAR*α* may also regulate the expression of FSP27/CIDEC as long-term treatment with fenofibrate, a PPAR*α* agonist, decreased hepatic FSP27/CIDEC expression and lipid content. Fenofibrate is widely used for the treatment of hypertriacylglycerolemia in patients with T2D. Although conflicting results have been reported in human studies [31, 32], data from our study and other studies have shown that fenofibrate treatment in rodents improved insulin resistance and fat deposition in the liver [33, 34]. As the major target tissue of fenofibrate activity, the liver may be responsible for the improved insulin sensitivity observed in fenofibrate-treated animals exhibiting lipid accumulation. As shown in our study and other studies, fenofibrate prevented HFD-induced hepatic TG accumulation and insulin resistance [35, 36]. However, the detailed mechanisms remained largely unknown. Our finding of fenofibrate-induced FSP27/CIDEC downregulation suggested a new mechanism for FSP27/CIDEC regulation and improved our understanding of the mechanism of PPAR*α*-dependent improvement in symptoms of FL. However, the negative regulation of FSP27/CIDEC by fenofibrate may be indirect as the PPAR*α* agonist Wy-14643 did not activate the functional peroxisome proliferator-activated receptor response element (PPRE) in the mouse* fsp27* promoter in HEK293 cells [8]. Moreover, forced expression of FSP27/CIDEC in hepatocytes significantly decreases the mitochondrial *β*-oxidation, which is negatively associated with TG accumulation in the liver [8]. Additionally, fenofibrate induces hepatic mitochondrial *β*-oxidation in obese rats [11]. These data suggest that fenofibrate increases hepatic mitochondrial *β*-oxidation by indirectly downregulating the expression of FSP27/CIDEC and ultimately decreasing hepatic lipid content. Therefore, it will be interesting to investigate the effects of fenofibrate on mitochondrial *β*-oxidation and lipid accumulation in the livers of liver-specific FSP27/CIDEC-knockout mice. During the preparation of this paper, a study published in* Hepatology* showed that the promoter of* FSP27β* (the major isoform in the liver) was activated by the liver-enriched transcription factor cyclic AMP-responsive element-binding protein H (CREBH) [18]. The* CREBH* promoter contains a functional PPPE site, which interacts with PPAR*α* [37]. CREBH physically interacts with PPAR*α* and regulates a variety of genes involved in fatty acid oxidation and hepatic lipid accumulation [38, 39]. Thus, further studies are required to investigate whether FSP27/CIDEC is involved in the interaction networks of CREBH and PPAR*α* in the liver.

In the present study, we observed for the first time that the expression of LSDP5 protein was markedly upregulated in FLs compared with normal livers in humans. LSDP5 is a newly identified member of the perilipin, ADFP, and TIP47 (PAT) family, which is ubiquitously expressed in tissues that exhibit high levels of fatty acid oxidation, including the heart, muscle, and liver [12, 40, 41]. In hepatocytes, LSDP5 is thought to contribute to TG accumulation by negatively regulating lipolysis and fatty acid oxidation [9]. However, the role of LSDP5 in hepatic steatosis in vivo is largely unknown. LSDP5 mRNA and/or protein levels are upregulated in different animal models with FL (e.g., rat fed an HFD, mice with FL dystrophy, and mice with *β*3 adrenergic receptor agonist-induced acute hepatic steatosis) [42–44]. Until now, no studies had described the hepatic expression of LSDP5 in humans. While our data support the hypothesis that LSDP5 protein expression is correlated with intrahepatic fat content in human subjects, more work is needed to confirm the association in a large sample population and to characterize its physiological and pathological roles in human hepatic lipid metabolism in future studies.

LSDP5 is modulated by both PPAR*α* and fasting in the mouse liver; thus, we investigated the regulation of LSDP5 protein expression by consumption of an HFD and treatment with PPAR (PPAR*α* and PPAR*γ*) agonists in mice. To our surprise, our data showed that LSDP5 expression was barely detectable in the livers of these mice, and neither HFD nor PPAR agonists induced the expression of this protein. This finding was somewhat inconsistent with other studies, in which LSDP5 protein was expressed in mouse liver [40, 43, 44]. The exact reasons for the discrepancy between our study and others are unknown. One possible explanation for the difference may result from the samples taken from the mice at different physiological conditions, in which our liver samples were obtained from fed mice, whereas other studies may be from fasted mice [43, 44]. Both PPAR*α* and fasting was reported to regulate the expression of LSDP5 in liver, and fasting statues itself induced LSDP5 mRNA expression independent of PPAR*α* activation in mouse liver [40]. Our data in the present study suggested that fasting/feeding might be a stronger factor for the expression level of LSDP5 than PPAR*α* in vivo. Future study might be needed to explore the possibility. Moreover, different animal models used in the studies may be another possibility for the differences [42–44].

In conclusion, we reported in the present study that the hepatic expression of FSP27/CIDEC and LSDP5 was upregulated in humans and that FSP27/CIDEC was increased in FLs of mice following consumption of an HFD. Long-term fenofibrate treatment decreased FSP27/CIDEC expression and hepatic TG content in mice fed an HFD. Our data suggest a potential new mechanism for FSP27/CIDEC expression in the liver and indicate a novel molecular mechanism of action of PPAR agonists, which should be further investigated.

## Supplementary Material

Supplemental materials includes one table and one figure in which present clinical background data of human liver samples (Table S1) and protein expression of hepatic LSDP5 in mice (Figure S1), respectively.
Supplementary Figure S1. LSDP5 protein expression in mouse liver. Immunoblot analyses of LSDP5 protein expression in liver from mice fed with a chow or high-fat diet (HF) for 20 weeks, and from db/db mice. M: mouse muscle, positive control for LSDP5 expression.

## Figures and Tables

**Table 1:** Metabolic phenotypes in mice before and after treatment.

Variables	Time (week)	Chow	HF	HF-P	HF-F	db/db
Body weight (g)	0	16.3 ± 1.0	17.6 ± 1.2	15.1 ± 0.3	14.6 ± 0.3	20.9 ± 0.8^a*∗*b*∗*^
	8	24.4 ± 0.9	28.4 ± 0.4^a*∗∗*^	25.5 ± 0.2^b*∗*^	24.3 ± 1.1^b*∗*^	49.9 ± 1.2^a*∗∗*b*∗∗*^
	12	24.2 ± 0.6	27.2 ± 1.4^a*∗*^	26.5 ± 0.8	25.0 ± 0.9	57.6 ± 3.2^a*∗∗*b*∗∗*^
	16	23.1 ± 1.3	28.9 ± 1.3^a*∗∗*^	29.2 ± 0.7	26.5 ± 0.6	
	20	24.4 ± 0.9	28.4 ± 1.2^a*∗∗*^	29.0 ± 0.8	25.8 ± 0.8	

Fasting glucose (mmol/L)	0	6.1 ± 0.2	6.2 ± 0.2	6.6 ± 0.1	6.6 ± 0.1	8.2 ± 0.3^a*∗*b*∗*^
	8	6.2 ± 0.4	8.4 ± 0.4^a*∗∗*^	8.1 ± 0.5	7.9 ± 0.3	15.8 ± 2.2^a*∗∗*b*∗∗*^
	12	5.8 ± 0.4	8.4 ± 0.7^a*∗∗*^	6.1 ± 0.3^b*∗∗*^	6.1 ± 0.3^b*∗∗*^	11.3 ± 1.7^a*∗∗*b*∗∗*^
	16	5.4 ± 0.2	8.0 ± 1.4^a*∗*^	6.1 ± 0.2	5.8 ± 0.2	
	20	6.1 ± 0.5	10.7 ± 0.4^a*∗∗*^	9.2 ± 0.5	8.1 ± 0.6^b*∗∗*^	

Epididymal fat (g)	20 (12^†^)	0.32 ± 0.05	0.71 ± 0.10^a*∗∗*^	0.72 ± 0.05	0.37 ± 0.05^b*∗∗*^	2.15 ± 0.36

Subcutaneous fat (g)	20 (12^†^)	0.23 ± 0.03	0.53 ± 0.09^a*∗∗*^	0.78 ± 0.03	0.25 ± 0.03^b*∗∗*^	10.24 ± 1.63

Fasting serum insulin (ng/mL)	20 (12^†^)	0.78 ± 0.04	1.78 ± 0.23^a*∗∗*^	0.91 ± 0.14^b*∗*^	1.04 ± 0.14^b*∗*^	4.07 ± 0.77

Serum TG (mmol/L)	20 (12^†^)	1.06 ± 0.15	0.92 ± 0.14	1.24 ± 0.09	1.15 ± 0.11	1.54 ± 0.15

Serum TC (mmol/L)	20 (12^†^)	0.77 ± 0.08	1.48 ± 0.14^a*∗∗*^	0.97 ± 0.05^b*∗∗*^	1.08 ± 0.06^b*∗∗*^	1.89 ± 0.13

Liver TG (*µ*mol/g)	20 (12^†^)	7.0 ± 0.5	12.3 ± 0.7^a*∗∗*^	10.9 ± 1.1	7.5 ± 0.8^b*∗∗*^	17.3 ± 3.0

Liver TC (*µ*mol/g)	20 (12^†^)	6.0 ± 0.4	9.3 ± 1.4	7.2 ± 0.5	6.9 ± 0.7	14.6 ± 0.3

Data represent means ± SEM. Statistical significance of differences between groups was analyzed with one-way analysis of variance (ANOVA) followed by Bonferroni's test.

Chow: mice fed a standard chow diet; HF: mice fed a high-fat diet; HF-P: mice fed a high-fat diet and treated with pioglitazone; HF-F: mice fed a high-fat diet and treated with fenofibrate; TC: total cholesterol; TG: triglyceride.

^†^Time for db/db. ^a^Versus Chow, ^b^Versus HF; ^*∗*^ *P* < 0.05, ^*∗∗*^ *P* < 0.01; *n* = 7.
