# Supplementary material for: Modulation Effect of Peroxisome Proliferator-Activated Receptor Agonists on Lipid Droplet Proteins in Liver
Source: J Diabetes Res. 2015 Dec 7;2016:8315454. doi: 10.1155/2016/8315454 (PMC4684860; doi:10.1155/2016/8315454)
Supplement: Supplementary file 1 — Supplemental materials includes one table and one figure in which present clinical background data of human liver samples (Table S1) and protein expression of hepatic LSDP5 in mice (Figure S1), respectively. Supplementary Figure S1. LSDP5 protein expression in mouse liver. Immunoblot analyses of LSDP5 protein expression in liver from mice fed with a chow or high-fat diet (HF) for 20 weeks, and from db/db mice. M: mouse muscle, positive control for LSDP5 expression. [file 8315454.f1.pdf]

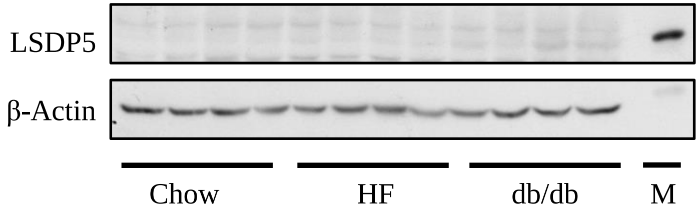

Supplementary Fig 1. LSDP5 protein expression in mouse fatty liver. Immunoblot analyses of LSDP5 protein expression in liver from mice fed with chow, high-fat diet (HF) for 20 weeks, and from db/db mice. M: mouse muscle, positive control for LSDP5 expression.

Supplementary Table 1 Clinical characteristic of subjects

Variables	Non-FL n = 4	FL n = 4
BMI (kg/m^2)	22.3 ± 0.5	26.3 ± 1.6
Age (y)	46 ± 5	43 ± 6
Triglyceride (mmol/L)	0.4 (0.3-1.0)	1.6 (0.9-2.3)
Total cholesterol (mmol/L)	2.0 (1.4-2.5)	3.7 (1.7-4.5)
Steatosis (%)	< 5	20-30

Data represent means ± SE for age and BMI, or median (min-max), or range.
